# Root Fracture and Extrusive Luxation in Primary Teeth and Their Management: A Case Report

**DOI:** 10.3390/dj9090107

**Published:** 2021-09-11

**Authors:** Gianni Di Giorgio, Giulia Zumbo, Matteo Saccucci, Valeria Luzzi, Gaetano Ierardo, Roberto Biagi, Maurizio Bossù

**Affiliations:** 1 Department of Oral and Maxillofacial Science, “Sapienza” University of Rome, Via Caserta, 6, 00161 Rome, Italy; gianni.digiorgio@uniroma1.it (G.D.G.); matteo.saccucci@uniroma1.it (M.S.); valeria.luzzi@uniroma1.it (V.L.); gaetano.ierardo@uniroma1.it (G.I.); maurizio.bossu@uniroma1.it (M.B.); 2 Department of Biomedical, Surgical and Dental Sciences, School of Dentistry, University of Milan, Via della Commenda 10, 20122 Milan, Italy; roberto.biagi@unimi.it

**Keywords:** dental trauma, root fracture, pulp injuries, orthodontic splint, primary tooth trauma, children

## Abstract

Background: Extrusion, lateral luxation, and intrusion are among the most serious types of dental trauma. Only a few studies have specifically focused on extrusion; the present one was aimed at reporting a case of domestic traumatic dental injury to primary tooth and describing the measures taken in managing the trauma in order to avoid future consequences to the underlying permanent tooth germ. Case report: A 3.5-year-old boy reported a dental injury with extrusion and root fracture of deciduous tooth 5.1. After intraoral and radiographic evaluation, the element was repositioned and stabilized by an orthodontic flexible splint attached to the adjacent teeth. Several follow-up checkups were made and showed good healing of the tissues and physiological exfoliation of the tooth, with a healthy and unaffected corresponding central permanent incisor. Conclusion: This case report strengthens the importance of well-timed diagnosis and treatment and of regular follow-up of traumatized teeth as they may affect both dentitions with a negative impact on Oral Health-Related Quality of Life. Conservative treatment should be taken into consideration when possible, being in some cases more appropriate.

## 1. Introduction

Traumatic dental injuries (TDIs) can cause problems related to teeth, as well as to their supporting tissues. These injuries can directly or indirectly affect the lives of the individuals, changing their appearance, speech, and position of teeth. TDIs in children and adolescents are considered serious health problems worldwide [1,2,3,4]. For children aged 0–6, oral injuries account for 18% of all physical injuries, and the mouth is the second most common involved area of the body [5]. A recent meta-analysis by Petti et al. on TDIs reported a 22.7% rate of TDIs affecting the primary teeth worldwide [6], with frequent recurrency of the trauma [7]. As children learn to crawl, walk, run, and discover the world around them, unintentional falls and collisions within leisure activities can often occur, becoming the most common causes of TDIs [8]. In fact, children aged between 2 and 6 are the most affected [5]. Increased overjet can be an important variable in increasing the risk of TDIs in the primary dentition [9]. Concerning primary dentition, luxation injuries are the most common TDIs, whereas, for permanent dentition, crown fractures are mainly reported [10].

Children with these injuries can turn to general dental practitioners, to emergency medical services, to pharmacists, to community dental clinics, or to specialist dental services. Therefore, appropriate knowledge, skills, and training in dealing with TDIs of primary dentition are required from each one of the abovementioned providers [5]. Proper diagnosis, treatment planning, and follow-up are important to assure a favorable outcome.

As reported by the latest International Association of Dental Traumatology (IATD) guidelines [10], examination and treatment in a young child are often difficult due to fear and lack of cooperation, causing distress for both the child and the parents. Treatment is not only conditioned by the child’s ability to cooperate, but also by the time occurring between the trauma and the professional intervention. In addition, in TDIs of deciduous teeth, the timing of exfoliation of the injured tooth plays a key role in treatment planning.

Moreover, the close relationship between the root apex of the traumatized primary tooth and the underlying permanent tooth germ always has to be taken into consideration and monitored. Tooth malformation, impacted teeth, and eruption disturbances in the developing permanent dentition can be consequences of severe injuries to primary teeth and/or alveolar bone.

Among the injuries, root fractures are rare (1–4%), and they affect the dentin, cementum, periodontal ligament, and pulp. They can be vertical, oblique, or horizontal, and diagnosis is obtained through clinical and radiographic examinations. The treatment depends on the diagnosis, as well as on the location [11,12]. Other injuries, such as extrusion, lateral luxation, and intrusion are some of the most severe types of dental trauma. In particular, extrusion has been reported to account for around 3% of all cases of traumatic dental injury, but only a few studies have focused on this type of injury [13,14].

Therefore, the present study was aimed at reporting a case of domestic traumatic dental injury to primary tooth, and describing the measures taken in managing the trauma in order to avoid future consequences to the underlying permanent tooth germ.

## 2. Case Report

**Figure 1:**
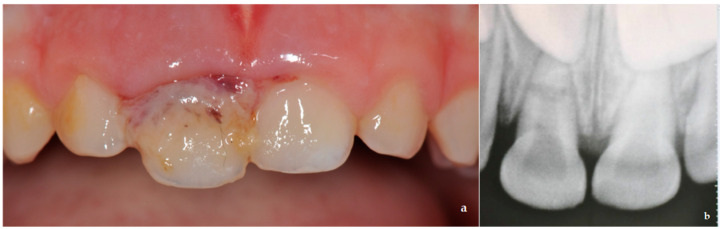
(**a**) Soft tissue lacerations with hematoma and a displacement of 3 mm along the axis of the deciduous maxillary right central incisor (5.1) were observed during intraoral examination; (**b**) periapical radiograph showed root fracture and absence of periodontal ligament (PDL) space alteration.

A 3.5-year-old boy with no relevant medical history was referred to the Pediatric Dentistry Unit of Policlinico Umberto I of Rome. Parents reported that the boy suffered a dental injury from a fall while playing at home. The child did not lose consciousness or vomit after the injury. Once informed consent from his parents was obtained, a medical anamnestic chart was filled, and a clinical exam was performed within 1 h of the trauma. During extraoral examination, no abnormalities of the temporomandibular joint were reported. Soft tissue lacerations with hematoma and a displacement of 3 mm along the axis of the deciduous maxillary right central incisor (5.1) were observed during intraoral examination. These resulted in a diagnosis of extrusion (Figure 1a). Furthermore, occlusal interference due to extrusion of the incisor prevented the molars from a proper occlusion. A periapical radiograph of the anterior teeth did not show altered periodontal ligament space, but horizontal root fracture was diagnosed (Figure 1b).

According to the protocols proposed by the IADT [5,10], the degrees of displacement and mobility, the root formation, the alteration of PDL space, and the child’s ability to deal with an emergency should be considered when planning the treatment. In cases of extrusion equal to or less than 3 mm, the protocol suggests careful repositioning or waiting for spontaneous repositioning. In cases of extrusion more than 4 mm, the tooth should be extracted. Furthermore, for root fracture with increased mobility and displacement, the protocol also suggests placement of orthodontic splinting according to the degree of mobility and compliance of the patient, in addition to periodic follow-up appointments.

**Figure 2:**
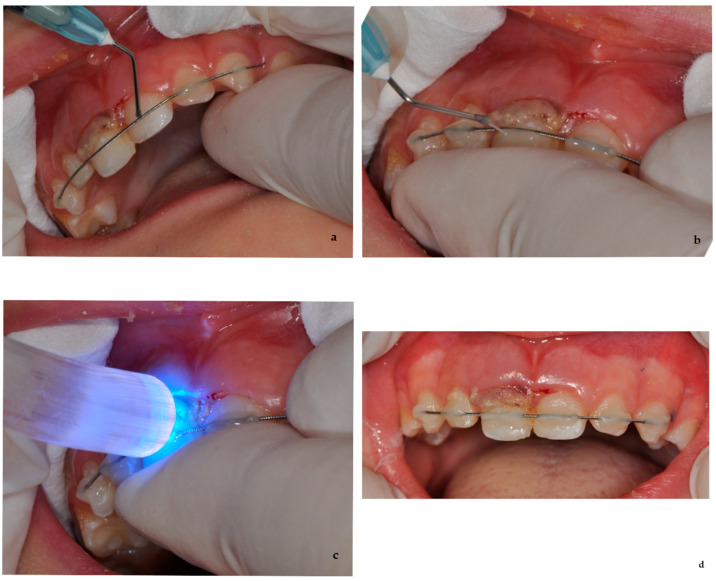
Different steps of the performed treatment: (**a**) preparation of the splint on the adjacent teeth; (**b**) gentle reposition of the element 5.1; (**c**,**d**) stabilization of the tooth through the splint.

Therefore, in the reported case, a thorough evaluation was carried out, and treatment was planned. The oral cavity was rinsed with 0.12% chlorhexidine solution, and local anesthesia was performed. An orthodontic flexible splint was prepared to be attached to the adjacent unaffected teeth (Figure 2a); then, the tooth was gently repositioned (Figure 2b), in order to avoid occlusal interference, and stabilized through the splint (Figure 2c,d).

A good prognosis following an oral trauma depends on effective oral hygiene; therefore, parents were instructed on how to take care of the injured tooth and prevent further injury by supervising potentially hazardous activities. They were instructed not to further traumatize the injured tooth when eating; nonetheless, a return to normal function as soon as possible was encouraged. Parents were advised to use a soft brush or cotton swab combined with alcohol-free chlorhexidine gluconate (0.12%) applied topically twice a day, in order to prevent accumulation of debris and to reduce the bacterial load. They were advised about possible complications that might occur, such as increased mobility, swelling, or a sinus tract; children might not complain about pain, but infection might still occur. If that happens, the child should be taken to a dentist for treatment.

In the reported case, a follow-up clinical examination was made 1 week after the injury, and the tooth was asymptomatic; there was normal color of the crown and lack of any signs of pulp necrosis and infection. The same follow-up appointment was planned for 4 weeks after the trauma for splint removal, if supported by healing signs. Aspects to be examined when carrying out a splint removal are color, symptoms, and mobility of the tooth, as well as healthy surrounding tissues. In this case, element 5.1 was asymptomatic; realignment of the root-fractured tooth was observed, mobility was physiological, and crown had a normal color. Supporting tissues were clinically healthy, and no signs of pulp necrosis or infection were observed. Thus, the splint was removed, and another follow-up appointment was planned for 8 weeks after the trauma. The same conditions and signs were observed at the subsequent checkups.

**Figure 3:**
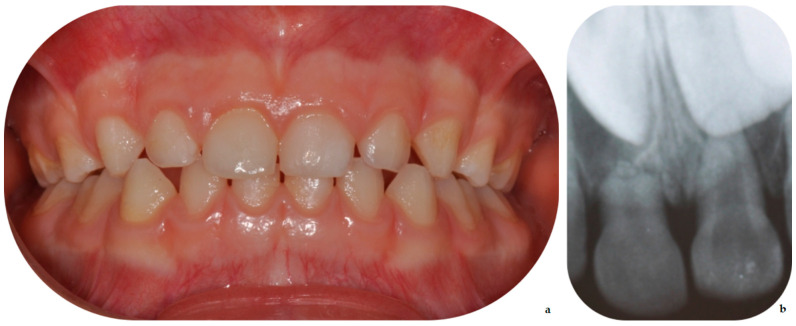
Good healing of the traumatized tooth and supporting tissues (**a**); in the radiograph (**b**) early signs leading to PCO and resorption of the radicular fragment.

At the 1-year follow-up appointment, the deciduous tooth showed no sensitivity to percussion nor pulp alterations. Tooth-supporting soft tissue was intact, and no pain or discomfort was reported. The crown showed no signs of alteration in color (Figure 3a). An anterior periapical radiograph was taken, and early signs leading to pulp canal obliteration (PCO) were present; PCO often occurs after dental injuries and can be associated or not with crown discoloration. No signs of pulp necrosis were present. Another aspect that can be observed in the periapical radiograph (Figure 3b) is the atypical resorption of the apical fragment, where—even if different from the physiological one–its characteristics are also different from pathologic root resorption.

**Figure 4:**
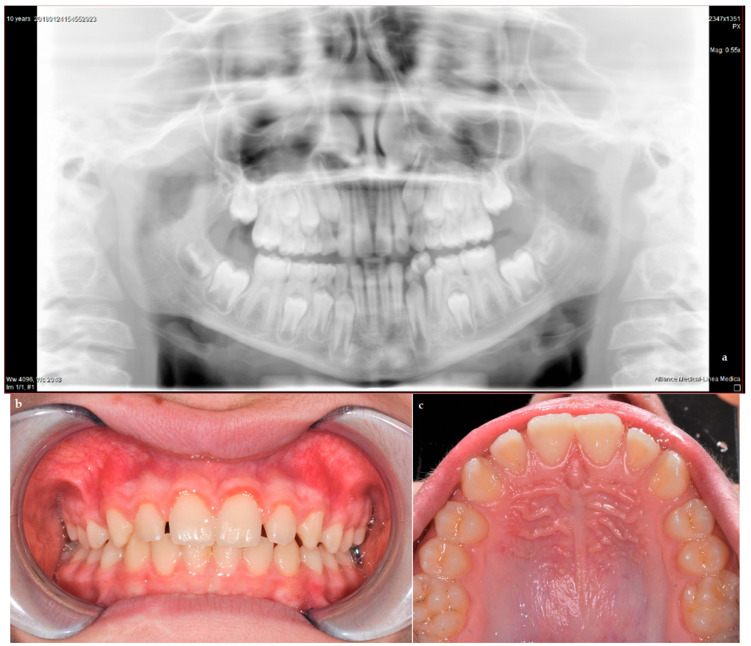
Panoramic view (**a**) shows normal development of the permanent successor at age 10; clinical pictures (**b**,**c**) show successful follow-up at age 12.

Frequent follow-ups were scheduled until the eruption of the permanent teeth; no abnormal changes were observed. After 3 years, when the child was 6.5, the deciduous maxillary right central incisor exfoliated and was physiologically replaced by the permanent right maxillary incisor (element 1.1). In the subsequent checkups, normal development of element 1.1 was observed, with no alteration in color or shape, demonstrating effective management of the trauma. The panoramic view in Figure 4a shows the follow-up at age 10, while Figure 4b,c show the clinical view at age 12.

## 3. Discussion

The main purposes of the diagnosis and management of TDIs in children with primary dentition are pain relief, preventing possible damage to the developing permanent germ, and minimizing the possibilities of sequelae [15,16]. TDIs can have a great influence on Oral Health-Related Quality of Life (OHRQoL) perceived by children and adolescents [17,18,19,20]. The literature reports cases evaluating sequelaes on permanent dentition after TDIs involving support tissue on the primary dentition [21,22,23]. The most critical period for the development of disorders in central incisors ranges from 4 months to 4 years of age. An earlier age of trauma results in a greater influence on treatment. Therefore, primary dentition should be clinically and radiographically monitored to detect alterations [23,24,25].

TDIs prognosis is difficult to predict. As a result, cost and quality-of-life implications should be considered when choosing treatment options. TDI treatment in deciduous teeth is different from that in permanent dentition; the close proximity of the root apex of the deciduous tooth to the permanent successor should be thoroughly considered. Inappropriate treatment of TDIs of primary teeth can cause greater damage than the trauma itself. Therefore, treatments preventing future sequelae of TDIs are decisive. According to IATD protocols, the degree of tooth displacement, tooth mobility, root formation, and the child’s ability to cope with an emergency should be considered by clinicians [16,18].

In this case, our team followed the protocols from the latest IATD guidelines [5,10], arranging a combination of the indications proposed for extrusive luxation and root fractures; the tooth was gently repositioned and splinted for 4 weeks to the adjacent ones. However, there is one case in the literature, described by Faria et al., where a more conservative treatment was chosen, still with successful results [16]. The latter regarded extrusive luxation in the primary central incisor; on the other hand, in our case, root fracture had to be considered in addition to extrusion, and a minimally invasive approach, similar to the one proposed by Faria et al., could not be proposed. Nevertheless, for future cases of extrusion comparable to that of Faria et al., a conservative approach could be taken into consideration, even if extraction would be suggested by the guidelines.

In light of this, it has to be considered that, when a periapical lesion occurs as a consequence of TDIs, such as the lateral luxation reported by Abreu et al. [16], pulpectomy can be useful to maintain and preserve the health of the teeth that would have otherwise been extracted [18,26]. This treatment aims at preserving normal periradicular tissue, preventing the progression of infection, and restoring the tooth to its function in the dental arch [27]. It was observed that treatment improved the OHRQoL of the child, as reported in other studies [28,29].

On the other hand, Cunha et al. [30] conducted a retrospective study on different types of treatment of TDIs in the deciduous dentition. The study included 315 children aged 1 to 4 years who previously suffered from TDIs. They reported cases of severe trauma, where invasive treatments were preferred, usually 6 months after the injury. Despite that, it was concluded that careful monitoring is the preferred treatment choice for TDIs in the deciduous dentition, even in severe cases. In fact, cases of success with procedures of minimal intervention for the management of TDIs in the deciduous dentition were reported by several authors. Conservative management successfully reduced iatrogenic complications and sequelae in the permanent dentition; these were mostly cases regarding the management of lateral luxation and intrusion [3,16,31,32,33].

Of course, the severity of sequelae in the permanent successor caused by trauma depends on factors such as the type of trauma to the primary tooth, the age of the child, the treatment that was done, and the direction of tooth displacement [34,35]. It is difficult to completely prevent accidents that might result in dental injuries. However, the complications from such accidents can be avoided with timely and adequate treatment and follow-up.

Regarding IATD recommendations for the treatment of root fractures and extrusions, splinting is strongly recommended. Despite that, and despite it being used and reported by authors, there are not many studies exploring the topic, and the prognosis related to splint use has not been fully investigated. Cho et al. [36] retrospectively investigated the outcomes of traumatic injuries in primary teeth treated with splinting. Among their conclusions, splinting in root fractures resulted in a better prognosis, confirming how this type of conservative treatment, together with prompt intervention, can favor a good outcome.

In the reported case, splinting proved to be effective, resulting in good outcomes. As previously stated, at 1 year follow-up, the deciduous tooth showed no sensitivity to percussion nor pulp alteration, and the crown showed no signs of alteration in color. Common signs of early pulp canal obliteration (PCO) were present; this often occurs after dental injuries and can be associated or not with crown discoloration [37,38]. It is important to remark that the work by Santos et al. [37], analyzing 112 traumatized teeth with 9 years follow-up, stated that there was no association between PCO and secondary pulp necrosis. Another aspect that can be observed in the periapical radiograph of the current case report, as well as in similar cases [39], is the atypical resorption of the apical fragment, where—even if different from the physiological one—its characteristics are also different from pathologic root resorption.

This case report is the first recent report regarding combined conservative management of extrusion and root fracture in deciduous dentition. This report concerns a conservative procedure with an extremely successful outcome. The correct diagnosis and treatment were crucial for the successful management and preservation of the traumatized teeth, avoiding any kind of sequelae. Nevertheless, further research is needed to ensure that clinicians have sufficient information on minimally invasive techniques to manage TDIs in deciduous dentition.

## 4. Conclusions

This case report strengthens the importance of well-timed diagnosis, prompt treatment, and regular follow-up of traumatized teeth, as a negative outcome may affect both dentitions, resulting in a possible negative impact on Oral Health-Related Quality of Life.

Furthermore, it should be highlighted that TDIs in primary teeth have to be taken seriously into consideration because they may result in damage to the underlying permanent tooth germ. A successful prognosis without damage for the permanent successor can be the result of proper treatment and checkups. Conservative treatment should be taken into consideration when possible, being in some cases more appropriate; therefore, inappropriate treatment of TDIs in the deciduous dentition can cause greater damage than the trauma itself. In conclusion, this case reinforced the importance of conducting regular follow-ups of injured teeth and underlined the concept that a prompt treatment can result in a good prognosis both for the deciduous tooth and for the permanent one.

## Data Availability

Not applicable.
